# Melatonin enhances SIRT1 to ameliorate mitochondrial membrane damage by activating PDK1/Akt in granulosa cells of PCOS

**DOI:** 10.1186/s13048-021-00912-y

**Published:** 2021-11-11

**Authors:** Bo Zheng, Junan Meng, Yuan Zhu, Min Ding, Yuting Zhang, Jianjun Zhou

**Affiliations:** Reproductive Medicine Center, Nanjing Drum Tower Hospital, Affiliated Hospital of Nanjing University Medical School, Zhongshan Road 32, Nanjing, 210008 China

**Keywords:** Melatonin, PCOS, Akt, SIRT1, mPTP

## Abstract

Mitochondrial injury in granulosa cells (GCs) is associated with the pathophysiological mechanism of polycystic ovary syndrome (PCOS). Melatonin reduces the mitochondrial injury by enhancing SIRT1 (NAD-dependent deacetylase sirtuin-1), while the mechanism remains unclear. Mitochondrial membrane potential is a universal selective indicator of mitochondrial function. In this study, mitochondrial swelling and membrane defect mitochondria in granulosa cells were observed from PCOS patients and DHT-induced PCOS-like mice, and the cytochrome C level in the cytoplasm and the expression of BAX (BCL2-associated X protein) in mitochondria were significantly increased in GCs, with p-Akt decreased, showing mitochondrial membrane was damaged in GCs of PCOS. Melatonin treatment decreased mitochondrial permeability transition pore (mPTP) opening and increased the JC-1 (5,5′,6,6′-tetrachloro1,1′,3,3′-tetramethylbenzimidazolylcarbocyanine iodide) aggregate/monomer ratio in the live KGN cells treated with DHT, indicating melatonin mediates mPTP to increase mitochondrial membrane potential. Furthermore, we found melatonin decreased the levels of cytochrome C and BAX in DHT-induced PCOS mice. PDK1/Akt played an essential role in improving the mitochondrial membrane function, and melatonin treatment increased p-PDK 1 and p-Akt in vivo and in vitro. The SIRT1 was also increased with melatonin treatment, while knocking down SIRT1 mRNA inhibiting the protective effect of melatonin to activate PDK1/Akt. In conclusion, melatonin enhances SIRT1 to ameliorate mitochondrial membrane damage by activating PDK1/Akt in granulosa cells of PCOS.

**Supplementary Information:**

The online version contains supplementary material available at 10.1186/s13048-021-00912-y.

## Introduction

Polycystic ovary syndrome (PCOS) is a common endocrine disorder affecting reproductive age women [1–4]. Excessive clinical or biochemical levels of androgen, ovulatory dysfunction, and polycystic ovarian morphology are recognized as the main characteristics of PCOS [5, 6]. Granulosa cells (GCs) play a vital role in hormone-secreting and follicle development and maturation [2]. Recently, studies showed that the content of mtDNA (mitochondrial DNA) in granulosa cells of PCOS patients was lower than the control group; meanwhile the expression of OXPHOS-related genes was down-regulated in PCOS patients [7, 8], which indicated that mitochondrial injury in GCs is associated with the pathogenesis of PCOS. The mechanism of mitochondrial injury in PCOS remains unclear. Some studies showed mitochondrial injury might be associated with excessive oxidative stress [5, 7]. However, study showed that mitochondrial membrane potential is a universal selective indicator of mitochondrial function, and is essential for mitochondrial ability to maintain a balanced metabolic micro-environment [9], indicating that mitochondrial membrane damage may leads to mitochondrial injury in GCs of PCOS patients.

Melatonin, centrally synthesized by the pineal gland, is ubiquitous molecules that involves synchronizing circadian rhythm and control of inflammation, ROS scavenging and immune response [10]. PCOS patients undergoing IVF-ET (in vitro fertilization embryo transfer) were found to present a significantly lower follicular melatonin concentration than that in the controls, which indicates a decrease of follicular melatonin concentration might result in characteristics of PCOS [11]. Our previous study found that melatonin treatment could ameliorate mitochondrial injury by enhancing SIRT1 in the GCs of PCOS, which further improve the phenotype of DHT-treated KGN cells and PCOS-like mice model [12]. However, whether melatonin ameliorates mitochondrial membrane damage by increasing SIRT1 remains unclear.

PDK1/Akt (phosphoinositide dependent proteinkinase-1/Protein Kinase B) signaling pathway is one of the major pathways to stabilize mitochondrial membrane potential and prevent mitochondrial membrane defect by decreasing mitochondrial permeability transition pore (mPTP) opening. Opening of mPTP can lead to mitochondrial swelling and cell death through apoptosis or necrosis [13]. In this study, we investigated whether melatonin enhances SIRT1 to ameliorate mitochondrial membrane damage in GCs of PCOS by activating PDK1/Akt.

## Methods

### Human sample collection

Human samples including ovarian granulosa cells and venous blood were collected at Drum Tower Hospital Affiliated to Nanjing University Medical School from 2019 to 2020. According to the 2003 Rotterdam criteria [14], the PCOS diagnosis with a hyperandrogenic phenotype included the following features, after excluding diseases such as congenital adrenal hyperplasia and androgen-secreting tumors: ovarian polycystic changes, oligo-ovulation or anovulation and hyperandrogenism. Patients requiring assisted reproductive treatment and younger than 35 years old were included. Infertile patients who have common ovulation with tubal factors were included to serve as controls. Human ovarian granulosa cell and blood samples were collected from both groups (*n* = 12 for each). Blood was collected by venous blood collection and serum were stored at − 80 °C. Analysis of serum hormones were performed using validated automated immunoassay methods (Elecsys electrochemiluminescence immunoassays on Cobas 6000, Roche Diagnostics).

The Institutional Review Board of the Drum Tower Hospital Affiliated to Nanjing University Medical School has approved this study. Informed written consent was provided by all participants.

### Transmission electron microscopy (TEM)

TEM sample preparation of human and mouse ovarian granulosa cells were conducted in Analysis and Detection Center of Nanjing Medical University. Briefly, cell samples were firstly washed with PBS and then fixed with 3% glutaraldehyde and 1% osmium acid. After dehydration with isoamyl acetate, samples were dried and treated with vacuum spraying to be conductive. Prepared samples were then examined using a FEI Tecnai G^2^ 20 TWIN transmission electron microscope (USA).

### Granulosa cell isolation and culture

After oocyte retrieval of patients, the human cumulus oophorus were digested with 0.1% hyaluronidase (Sigma-Aldrich, USA) for 2 min. Then DMEM/F12 (Gibco BRL/Invitrogen) containing 10% newborn calf serum (Gibco BRL/Invitrogen) was used to end digestion. Samples of human granulosa cells were then separated by centrifugation at 500 g in a swing bucket centrifuge for 35 min. After isolation, incubation of human GCs and KGN cells was conducted in DMEM/F12 (Gibco BRL/Invitrogen) containing 10% newborn calf serum (Gibco BRL/Invitrogen) and 1% (v/v) penicillin/streptomycin as previously reported [12]. The KGN cell line was a generous gift from Dr. Yiming Mu at the General Hospital of the People’s Liberation Army, Beijing, China [15]. Briefly, all cells were cultured in a 5% CO_2_ incubator at 37 °C. KGN cells would be treated with 500 nM DHT (MCE, USA) with or without 100pM melatonin (Sigma-Aldrich, USA) for 24 h in certain experiments.

### Western blotting

Total protein extraction was conducted as previously reported [15]. A mitochondrial isolation kit was purchased for (GENMED, China) mitochondrial protein isolation. Twenty micrograms of total protein or mitochondrial protein from cells, mice tissues and human samples were resolved and then transferred to PVDF membranes. Afterwards, 5% blotting-grade blocker (Bio-rad, USA) or BSA albumin fraction V (Biofroxx, USA) was provided for 90 min to block the PVDF membrane. Primary antibodies for immunoblotting against SIRT1 (1:10000, ab189494, Abcam), Akt (1:1000, 9272S, Cell Signaling), p-Akt (1:1000, 9271S, Cell Signaling), PDK1 (1:1000, 5662S, Cell Signaling), p-PDK1 (1:1000, AF3018, Affinity), cytochrome C (1:1000, ab133504, abcam), Bcl2 (1:1000, 12,789–1-AP, Proteintech), BAX (1:1000, ab32503, abcam), VDAC (1:10000, CY5416, Abways) and β-actin (1:10000, ap0060, Bioworld Technology), the goat anti-rabbit HRP-conjugated secondary antibody was then incubated (1:10000, a0045, sigma). The antibodies used in our study have been previously validated in former works [15, 16]. Diaminobenzidine was used to detect protein signals and ImageJ software (NIH, Bethesda, MD, USA) was used to perform measurement of protein expression in a blind fashion.

### Animals

Twenty-four female C56BL/6 mice (3-week-old) were provided by the Experimental Animal Center at Nanjing Medical University (Nanjing, China). These mice were then raised on a 12-h light: 12-h dark cycle with available food and water in the Drum Tower Hospital Animal Laboratory Center (Nanjing, China).

Mice were randomly divided into vehicle group (*n* = 6) and DHT-induced group (*n* = 18). The treatment was given once a day, using subcutaneous injection containing 0.1 ml/100 (g.d) sesame oil (Sigma-Aldrich, USA) for the oil group or 6 mg/100 (g.d) DHT (MCE, USA) diluted in 0.1 ml/100 g sesame oil for the DHT-induced group as previously described [12]. Mice body weight was recorded for 35 consecutive days. Mice estrous cycles was determined by judging cell types of vaginal smears daily.

After the PCOS-like mice models were developed, six mice of vehicle group and six mice of DHT group were euthanized to collect the bilateral ovaries and blood at the diestrus stage.

The remaining twelve mice of DHT group were randomly divided into a control group (*n* = 6) and a melatonin group (n = 6). Melatonin (Sigma-Aldrich, USA) was dissolved in 0.9% saline containing 0.5% ethanol; 0.5% ethanol saline (v/v) is regarded as vehicle. Mice were treated with vehicle or melatonin (10 mg/kg) by oral gavage at 6:00 p.m. once a day (during this treatment, DHT was no longer given to mice) for 14 dyas [17]. Also, all mice were euthanized to collect blood and bilateral ovaries. Mouse granulosa cell isolation was conducted as previously reported [15]. And for the western blots from mice, samples were the whole ovaries.

### Estrous cycle analysis

Vaginal smears were taken from mice vagina with a glass rod daily. The estrous cycle stage was examined optically by microscope analysis of the vaginal epithelial cell smears.

### Histology and follicle counting

Mice ovaries were collected and cleaned. The cleaned ovary samples were then fixed with 10% formalin and cut into 5 μm slices, followed by stained with hematoxylin and eosin. Afterwards, an unbiased stereological method was used to count the follicles, and only the follicles with an oocyte nucleus were counted. The raw number of follicles was multiplied by five to determine the total number of follicles, as the sampling fraction with respect to every 5th section being counted. The follicles were classified according to the classification system used by previous studies [18].

### Serum hormone determination

Mice blood samples were collected and serum components were separated for mice serum hormone detection including testosterone (T), follicle stimulating hormone (FSH) and luteinizing hormone (LH) by ELISA kits (E0457m, Yanyu, Shanghai, China for T, E0441m, Yanyu, Shanghai, China for FSH and E0457m, Yanyu, Shanghai, China for LH) as our previous study [12].

### Measurement of the mitochondrial membrane potential

Mitochondrial membrane potential assay kit with JC-1 (Beyotime, China) was purchased to evaluate the mitochondrial membrane potential, the fluorescence intensity of the JC-1 monomers/aggregates (green fluorescence for monomer, red fluorescence for aggregate) were taken by fluorescence microscopy (Leica), following the instructions of JC-1 assay kit. Digital images were analyzed with ImageJ software (NIH, Bethesda, MD, USA). The calculation results of the red/green fluorescence ratio were for assessing mitochondrial membrane potential.

### Measurement of mitochondrial permeability transition pore (mPTP)

A mitochondrial Permeability Transition Pore Assay Kit (Beyotime, China) was purchased to detect the mitochondrial permeability transition pore. All procedures were performed following the instructions. Briefly, cells were incubated with staining solution for 30 min at 37 °C. After incubation, cells were cultured in DMEM/F12 medium (Gibco BRL/Invitrogen) for another 30 min and then resuspended for flow cytometry. For flowmetry, 488 nm argon ion laser was detected every 5 minutes for 30 min and total number of detected cells were over 50,000 per sample. Average optical density was evaluated for measuring mPTP.

### RNA interference

SIRT1 small interfering RNA (SIRT1 siRNA) was purchased from RiboBio (China). The siRNA transfection was performed according to the instruction with Lipo3000 (Invitrogen, USA). 24 h after the transfection, the KGN cells were cultured in medium with or without DHT and/or melatonin treatment for another 24 h.

### Immunofluorescence

After being fixed in cold 4 g/100 mL paraformaldehyde, the KGN cells were transferred to 5/100 mL, 10/100 mL, and 15/100 mL sucrose/PBS, followed by embedding in optimum cutting temperature compound (OCT; Sakura Finetek USA Inc.). The blocks were then frozen in liquid nitrogen. After three washes with PBS removing OCT, the slides were incubated in 1.5/50 mL BSA/PBS for 1 h in 37 °C and then incubated in antibodies. The positive signals were visualized by the fluorescence-conjugated secondary antibodies. The slides were mounted with mounting medium with DAPI (Vector Lab) and viewed using a fluorescence microscope (Leica). Antibodies against MTNR1A (1:500; A13030, ABclonal), MTNR1B (1:500; DF4994, Affinity) were used for immunofluorescence.

### Statistical analysis

The mean ± standard error of mean (SEM) of experimental variables were assessed. One-way ANOVA were performed to analyze the data from more than two groups and Students’ t-tests were performed to evaluated the statistical significance between two groups using GraphPad Prism 8 statistical software (San Diego, USA). *P* < 0.05 was considered as with statistical significance.

## Results

### Clinical characteristics

The clinical characteristics were compared between control and PCOS group (Table 1). Age (30.13 ± 2.54 vs 29.77 ± 2.47 years, *P* = 0.35), the basal FSH level (7.90 ± 1.49 vs 7.83 ± 2.44, 7.83, *P* = 0.54) or BMI (23.57 ± 2.58 vs 22.48 ± 2.04, *P* = 0.42) had no significant difference between these two groups, while basal T (0.31 ± 0.20 vs 1.08 ± 0.34, *P* < 0.01), the basal LH (4.37 ± 1.11 vs 17.05 ± 5.63, *P* < 0.01) and LH/FSH ratio (0.50 ± 0.21 vs 2.33 ± 0.83, *P* < 0.01) were increased in the PCOS group.

### Mitochondria membrane was damaged in the PCOS patients’ granulosa cells

**Fig. 1:**
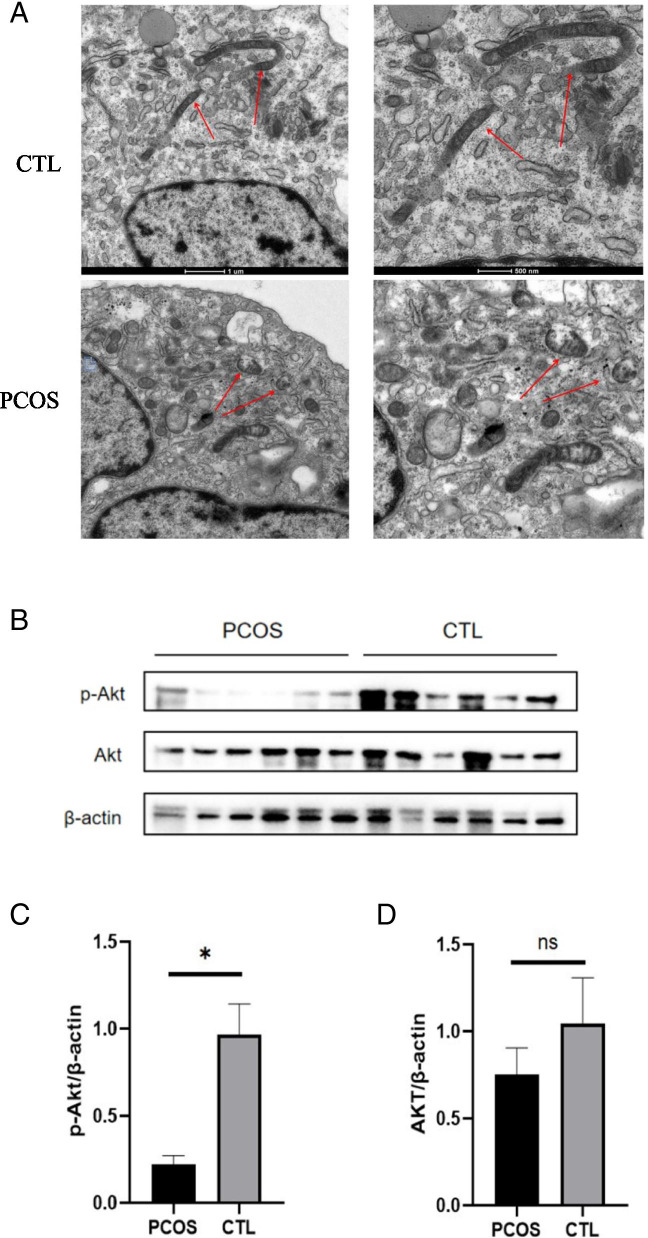
Mitochondria membrane damage was observed in the GCs of the PCOS patients. **A** Mitochondrial ultrastructure detected by TEM (*n* = 3 in each group). Mitochondrial swelling and mitochondrial membrane defect were observed in the PCOS group. The arrows point at mitochondria. **B-D** The p-Akt protein level was decreased in the PCOS patients while total Akt expression has no difference (*n* = 6 in each group). *P < 0.05, **P < 0.01, ***P < 0.001.

TEM (transmission electron microscopy) was applied to inspect the function of mitochondria membrane in the GCs. Mitochondrial swelling and mitochondrial membrane defect were found in the PCOS patients’ granulosa cells (Fig. 1A). Akt activation played a vital role in maintaining the mitochondrial membrane permeabilization (MMP), while the p-Akt level in GCs was decreased in PCOS patients (Fig. 1B-D, *n* = 6 for each group).

### Increased mitochondrial membrane damage in the DHT-induced PCOS mouse model

**Fig. 2:**
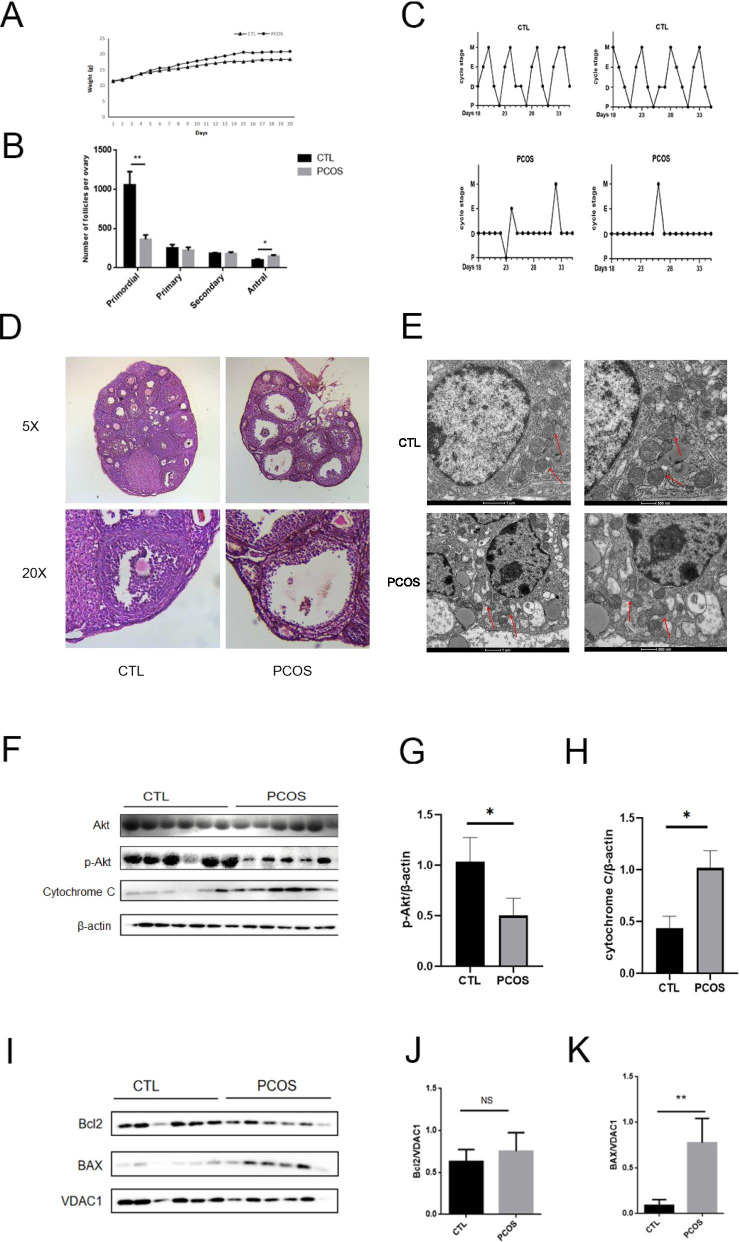
Mitochondrial membrane damage and decreased p-Akt expression in the DHT-induced PCOS mouse model. **A** Higher weight in the DHT-treated PCOS-like mice. (*n* = 6 in each group) **B** More antral follicles, less primordial follicles in the PCOS mice (n = 6 in each group) **C** Disrupted estrous cycles (mainly in diestrus) in the PCOS mice, while the control mice had normal estrous cycles (n = 6 in each group) **D** Polycystic ovary morphology with fewer corpus lutea and thinner layers of granulosa cells were found in the PCOS mice ovaries by optical microscopy (n = 6 in each group) **E** TEM revealed mitochondria swelling and mitochondrial membrane defect in the GCs of the PCOS mice. The arrows point at mitochondria. (*n* = 3 in each group) **F-H** We evaluated the p-Akt and cytochrome C expression in the ovaries of PCOS mice. The level of p-Akt protein was significantly decreased in the PCOS mice while cytochrome C expression of increased. (n = 6 in each group) **I-K**. BAX, Bcl2, and VDAC1 expression in the mitochondria were evaluated, and BAX level was significantly higher in the PCOS mice. (n = 6 in each group) NS, not significant, *P* > 0.05, *P < 0.05, **P < 0.01, ***P < 0.001, ****P < 0.0001

DHT-induced PCOS mice showed disrupted estrous cycles and higher weight, while the mice in control group had a normal estrous cycle of 4–5 days (Fig. 2A, C), along with increased serum level of testosterone and FSH/LH ratio [12]. The DHT treated mice also showed fewer CLs, more antral follicles, less primordial follicles, and vivid ovarian cyst expansion and thinner layers of granulosa cells (Fig. 2B, D). The mitochondrial swelling and defect of mitochondrial membrane were also presented in the GCs of the PCOS-like mice by TEM (Fig. 2E). Usually, mitochondria begin to swell till membrane defect occurred and let out cytochrome C after the increase in organelle osmotic pressure led by MMP [13]. Additionally, we evaluated p-Akt and cytochrome C expression in mice GCs after DHT treatment. The cytochrome C protein level was significantly increased and the p-Akt level was decreased in PCOS mice when compared to those levels expressed in control group (Fig. 2F-H). Besides, we detected BAX expression in mitochondria in the GCs and found that BAX protein level was increased in PCOS mice (Fig. 2I-K), which reflected decreased mitochondrial membrane potential stability. These results showed that the mitochondrial membrane was damaged in the DHT-induced PCOS mice.

### Melatonin increases SIRT1 expression and ameliorates mitochondrial membrane damage in the KGN cells treated with DHT

**Fig. 3:**
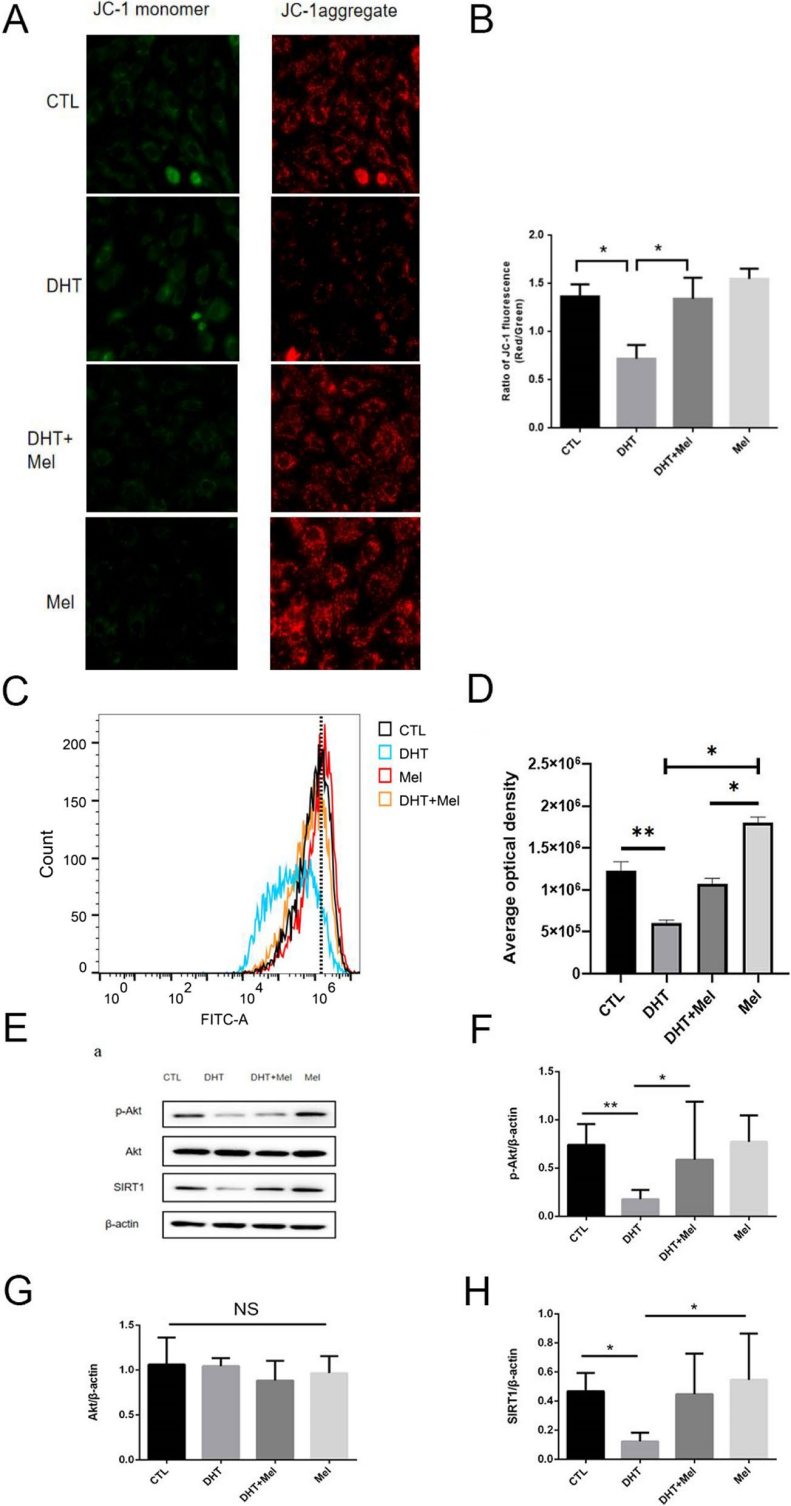
Melatonin increases mitochondrial membrane potential and promotes p-Akt expression in the DHT-treated KGN cells. **A-B** Level of mitochondria membrane potential in the DHT group was lower than the control group, and melatonin treatment attenuated the decrease. Ratio of JC-1 aggregate/JC-1 monomer was measured for mitochondrial membrane potential. (*n* = 3 in each group) **C-D** Level of mPTP AOD (average optical density) was also significantly decreased in the KGN cells and increased after treated with melatonin. (n = 3 in each group) **E-H** The level of p-Akt and SIRT1 protein expression was decreased when treated with DHT, and melatonin treatment attenuated this change. (n = 3 in each group) NS, not significant, *P* > 0.05, *P < 0.05, **P < 0.01

The SIRT1 protein expression was downregulated in in DHT-treated KGN cells, when co-treated with melatonin, the SIRT1 expression was increased (Fig. 3H). In order to study whether melatonin mediates the mitochondrial membrane function, the ratio of JC-1 aggregate/monomer in fluorescence of live KGN cells was recorded to measure mitochondrial membrane potential (Fig. 3A-B), and the mitochondrial permeability transition pore (mPTP) opening of live KGN cells was measured by flow cytometry (Fig. 3C-D). P-akt could suppress excessive mPTP opening to improve mitochondrial membrane potential. Thus, we evaluated p-Akt level as well. The increased mPTP opening and decreased JC-1 aggregate/monomer ratio was observed with DHT treatment, while melatonin treatment decreased mPTP opening and increased JC-1 aggregate/ monomer ratio, and the p-Akt level was also increased (Fig. 3E-G). Our results suggested that melatonin treatment could increase the SIRT1 expression and decrease mPTP opening to arise mitochondrial membrane potential, and eventually ameliorate mitochondrial membrane damage in DHT-treated KGN cells.

### Melatonin attenuates mitochondrial membrane damage in the DHT-induced PCOS mice

**Fig. 4:**
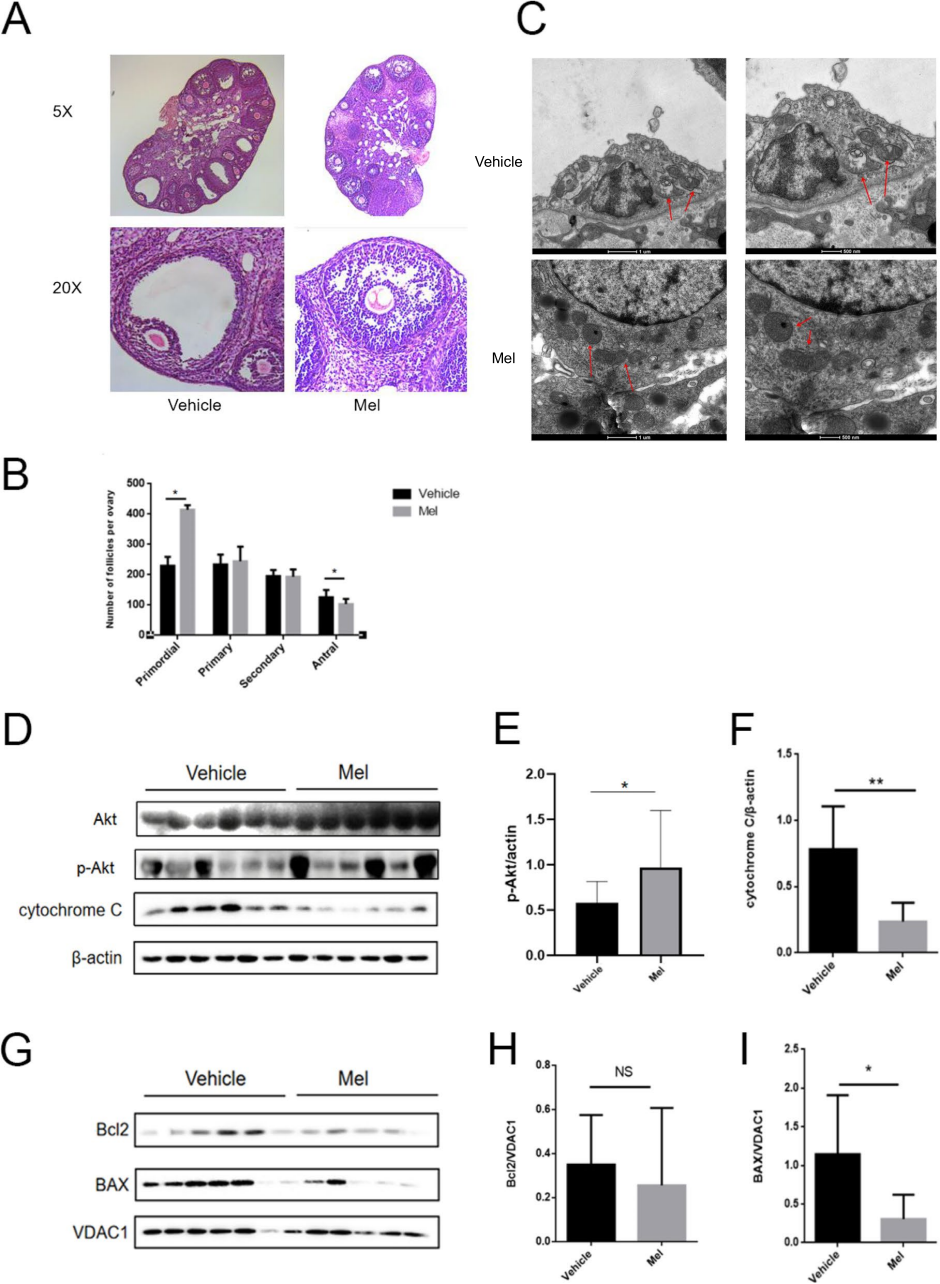
Melatonin attenuates the PCOS like phenotype and ameliorates mitochondrial membrane damage. **A** The PCOS-like microstructure in the DHT-treated mice was significantly attenuated after melatonin treatment. (n = 6 in each group) **B** Follicle counting revealed that melatonin-treated mice had fewer antral follicles and more primordial follicles when compared with the PCOS mice (n = 6 in each group). **D-F** the increasing expressions of cytochrome C and decreased expression of p-Akt were attenuated after melatonin treatment (n = 6 in each group) **G-I**. After treated with melatonin, BAX level was significantly decreased (n = 6 in each group) (N). NS, not significant, *P* > 0.05, *P < 0.05, **P < 0.01

To evaluate the effect of melatonin in maintaining the mitochondrial membrane potential in vivo, the DHT-induced PCOS mice were treated with melatonin. After melatonin treatment, the mitochondrial swelling and membrane defect in the PCOS mice was notably ameliorated (Fig. 4A), and follicle counting showed more primordial follicles and fewer antral follicles than the mice not treated with melatonin (Fig. 4B). Furthermore, less mitochondrial swelling and mitochondrial membrane defect were observed in the GCs of the melatonin-treated mice (Fig. 4C). Furthermore, the level of cytochrome C expression and BAX expression were decreased (Fig. 4D-I), while p-Akt expression was increased after melatonin treatment. The results indicated that melatonin could attenuate the PCOS phenotypes and mitochondrial membrane damage in the GCs of the DHT-induced PCOS mice.

### Melatonin enhances SIRT1 to ameliorate mitochondrial membrane damage by activating PDK1/Akt

**Fig. 5:**
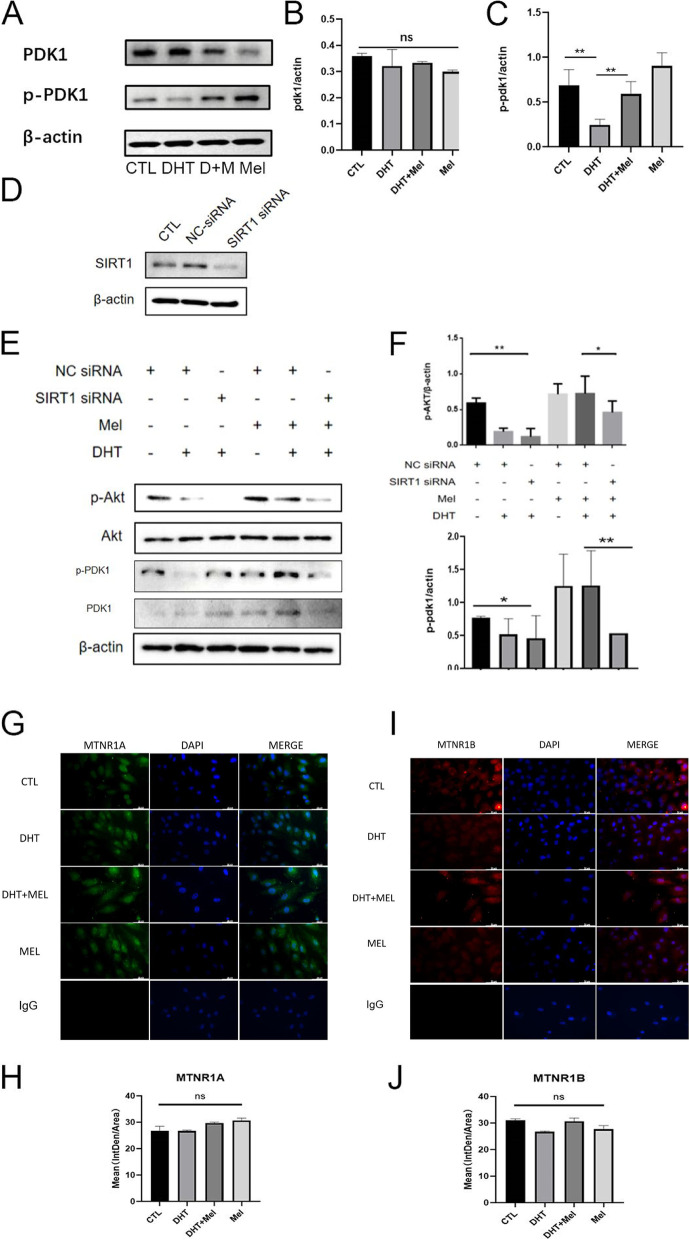
Effects of SIRT1 on Akt activation in KGN cells. **A-C** DHT treatment decreased p-PDK1 expression, and melatonin attenuated this change. While total PDK1 expression showed no significant difference. (n = 3 in each group) **D** SIRT1 was knocked down by SIRT1 siRNA. (n = 3 in each group) **E-F** Melatonin treatment failed to increase the p-PDK1 expression and activated Akt level in the SIRT1 siRNA transfected KGN cells. (n = 3 in each group) **G-J** Melatonin receptors MTNR1A and MTNR1B expression showed no significant difference in the KGN cells by fluorescent microscopy. (n = 3 in each group) *P < 0.05, **P < 0.01, ***P < 0.001, ****P < 0.0001

p-PDK1 is a necessary molecular for Akt activation. The p-PDK1 level was decreased in DHT-treated KGN cells, resulted in p-Akt decrease, however, melatonin reversed these changes (Fig. 5A-C). In order to investigate whether melatonin attenuated mitochondrial membrane damage in GCs by improving SIRT1 level to activate PDK1/Akt, firstly we used SIRT1 siRNA to knock down SIRT1 in KGN cells (Fig. 5D). Melatonin treatment failed to increase p-PDK1 or p-Akt level in the SIRT1 knocked-down cells, suggesting that melatonin may attenuate mitochondrial membrane damage through SIRT1-PDK1-Akt (Fig. 5E-F).

We further investigated whether melatonin treatment affected its receptors MTNR1A and MTNR1B, and the immunofluorescence showed that the effect of attenuating mitochondrial membrane damage was not through change the level of MTNR1Aor MTNR1B (Fig. 5G-J).

## Discussion

This study showed that mitochondrial membrane was damaged the GCs of PCOS patients, and melatonin might activate PDK1/Akt by promoting SIRT1 expression to ameliorate mitochondrial membrane damage in GCs of PCOS.

Mitochondria function as one of the most critical intracellular redox metabolism and reactive oxygen species producer [19]. The complete structure of mitochondrial membrane is essential for formal mitochondrial function. To maintain primary biological function of mitochondria, PI3K/Akt signaling pathway is one of the major pathways to stabilize mitochondrial membrane potential and prevent mitochondrial membrane defect by decreasing mPTP opening. After mitochondrial membrane defect occurs, mitochondria swelling was observed, and more cytochrome C was released, which enhanced apoptosis cascade and led to cell death [20]. Many studies have proved the release of cytochrome C as a vital event of apoptosis so that the level of released cytochrome C could be targeted as a biomarker of mitochondrial damage [21–23]. BAX, a member of Bcl2 family which enhances the release of cytochrome C into the cytoplasm, can reflect mitochondrial function as well [13, 24, 25]. Our study found increased cytochrome C release, BAX expression and decreased p-Akt level in GCs of PCOS mice and PCOS patients, showing the essential role of mitochondrial membrane damage in PCOS pathogenesis. The main cause of the defective structure of mitochondrial membrane was abnormal mitochondrial membrane potential [26–28], which is in accordance with our results. As we have proved the excessive mPTP opening was observed in GCs of PCOS and mitochondrial membrane potential was significantly down-regulated, it was reasonable to presume the excessive mPTP opening might decreased mitochondrial membrane potential and eventually cause mitochondrial dysfunction.

Melatonin is a broad-spectrum antioxidant secreted by pineal gland, retina, gastrointestinal tract and skin. Studies have found that melatonin could preserve mitochondrial homeostasis under different experimental conditions because of its antioxidant and anti-inflammatory action [29]. Former studies show that melatonin has therapeutic effects on steroidogenesis, folliculogenesis and oocyte maturation in PCOS [30]. Recently, one study reported that melatonin could preserve proper mitochondrial membrane potential rather than remove ROS directly [28]. Our previous study showed that melatonin might promote SIRT1 expression to ameliorate mitochondrial injury in GCs of PCOS. This study found that with melatonin treatment, mitochondrial membrane potential was increased, and the PCOS-like phenotype of mice such as polycystic ovarian expansion was attenuated. Thus, we suspected that melatonin may improve the mitochondria function of PCOS patients by protecting GCs’ mitochondrial membrane potential.

Akt activation is a key event of PI3K/Akt signaling pathway which proved to be a major mediator of mitochondrial membrane potential and directly mediated by SIRT1 [31, 32]. A decrease in mitochondrial membrane potential was believed to be a significant appearance of mitochondrial injury [33]. Several studies confirmed deficient Akt activation could lead to mitochondrial dysfunction and injury [34–36]. In the current study, the mechanism of melatonin improving mitochondrial function in GCs of PCOS patients was highly related to the classic PI3k/Akt signaling pathway, which was also highly relevant to PCOS pathogenesis [37, 38]. Firstly, the expression of activated Akt, p-Akt, was significantly reduced in PCOS patients, PCOS mice, and DHT-induce KGN cells. Secondly, the key component of Akt complex -- PDK1 was also less activated in the PCOS group. Thirdly, melatonin treatment could increase the level of p-Akt and p-PDK1 protein, which led to a more stable mitochondrial membrane potential and mitochondrial function, while melatonin treatment failed to increase p-PDK1 or p-Akt level in the SIRT1 knocked-down cells. These results suggested that melatonin may attenuate mitochondrial membrane damage through SIRT1-PDK1-Akt. Furthermore, no significant change was showed in MTNR1A/MTNR1B after melatonin treatment, showing the protective effect of melatonin was not through melatonin receptor changes.

## Conclusions

In conclusion, mitochondrial membrane damage in GCs might be involved in the pathophysiological mechanism of mitochondrial injury in PCOS. Melatonin protects against mitochondrial membrane damage in GCs by enhancing SIRT1 expression to activate PDK1/Akt. These results may provide novel insights into the mechanism of melatonin ameliorates mitochondrial injury in the GCs of PCOS.

## Supplementary Information

**Additional file 1: Supplementary Figure 1**. Raw data of western blotting. **Supplementary Figure 2**. Cycle stages of all PCOS mice and control mice. **Supplementary Figure 3**. Expression of Beta-actin between PCOS and Control in Fig. 1

## Data Availability

All data generated or analysed during this study are included in this published article.

## Abbreviations

PCOS: Polycystic ovary syndrome
SIRT1: NAD-dependent deacetylase sirtuin-1
Akt: Protein Kinase B
PDK1: Phosphoinositide dependent proteinkinase-1
PIP3: Phosphatidylinositol-3-phosphate
mPTP: Mitochondrial permeability transition pore
BMI: Body mass index
FSH: Follicle-stimulating hormone
LH: Luteinizing hormone
T: Testosterone
DHT: Dihydrotestosterone
Mel: Melatonin
GCs: Granulosa cells
BAX: BCL2-associated X protein

## References

[1] Goodarzi MO, Dumesic DA, Chazenbalk G, Azziz R (2011). Polycystic ovary syndrome: etiology, pathogenesis and diagnosis. Nat Rev Endocrinol.

[2] Knochenhauer ES, Key TJ, Kahsar-Miller M, Waggoner W, Boots LR, Azziz R (1998). Prevalence of the polycystic ovary syndrome in unselected black and white women of the southeastern United States: a prospective study. J Clin Endocrinol Metab.

[3] Laganà AS, Garzon S, Casarin J, Franchi M, Ghezzi F (2018). Inositol in polycystic ovary syndrome: restoring fertility through a pathophysiology-based approach. Trends Endocrinol Metab.

[4] Taylor AE, McCourt B, Martin KA, Anderson EJ, Adams JM, Schoenfeld D (1997). Determinants of abnormal gonadotropin secretion in clinically defined women with polycystic ovary syndrome. J Clin Endocrinol Metab.

[5] Azziz R, Carmina E, Chen Z, Dunaif A, Laven JS, Legro RS (2016). Polycystic ovary syndrome, nature reviews. Dis Primers.

[6] Thackray VG (2019). Sex, microbes, and polycystic ovary syndrome. Trends Endocrinol Metab.

[7] Lee SH, Chung DJ, Lee HS, Kim TJ, Kim MH, Jeong HJ (2011). Mitochondrial DNA copy number in peripheral blood in polycystic ovary syndrome. Metabolism.

[8] Skov V, Glintborg D, Knudsen S, Jensen T, Kruse TA, Tan Q (2007). Reduced expression of nuclear-encoded genes involved in mitochondrial oxidative metabolism in skeletal muscle of insulin-resistant women with polycystic ovary syndrome. Diabetes.

[9] Singh R, Barden A, Mori T, Beilin L (2001). Advanced glycation end-products: a review. Diabetologia.

[10] Cipolla-Neto J, Amaral FGD (2018). Melatonin as a hormone: new physiological and clinical insights. Endocr Rev.

[11] Tamura H, Nakamura Y, Korkmaz A, Manchester LC, Tan DX, Sugino N (2009). Melatonin and the ovary: physiological and pathophysiological implications. Fertil Steril.

[12] Yi S, Zheng B, Zhu Y, Cai Y, Sun H, Zhou J (2020). Melatonin ameliorates excessive PINK1/Parkin-mediated mitophagy by enhancing SIRT1 expression in granulosa cells of PCOS. Am J Physiol Endocrinol Metab.

[13] Kroemer G, Galluzzi L, Brenner C (2007). Mitochondrial membrane permeabilization in cell death. Physiol Rev.

[14] Rotterdam ESHRE/ASRM-Sponsored PCOS consensus workshop group. Revised 2003 consensus on diagnostic criteria and long-term health risks related to polycystic ovary syndrome (PCOS). Hum Reprod. 2004;19(1):41–7. 10.1093/humrep/deh098. 10.1093/humrep/deh098 14688154

[15] Zhang M, Zhang Q, Hu Y, Xu L, Jiang Y, Zhang C (2017). miR-181a increases FoxO1 acetylation and promotes granulosa cell apoptosis via SIRT1 downregulation. Cell Death Dis.

[16] Ding L, Ding Y, Kong X, Wu J, Fu J, Yan G (2019). Dysregulation of Krüppel-like factor 12 in the development of endometrial cancer. Gynecol Oncol.

[17] Wu J, Zhu D, Zhang J, Li G, Liu Z, Sun J (2016). Melatonin treatment during the incubation of sensitization attenuates methamphetamine-induced locomotor sensitization and MeCP2 expression. Prog Neuro-Psychopharmacol Biol Psychiatry.

[18] Sheng X, Yang Y, Zhou J, Yan G, Liu M, Xu L (2019). Mitochondrial transfer from aged adipose-derived stem cells does not improve the quality of aged oocytes in C57BL/6 mice. Mol Reprod Dev.

[19] Zorov DB, Juhaszova M, Sollott SJ (2014). Mitochondrial reactive oxygen species (ROS) and ROS-induced ROS release. Physiol Rev.

[20] Santucci R, Sinibaldi F, Cozza P, Polticelli F, Fiorucci L (2019). Cytochrome c: an extreme multifunctional protein with a key role in cell fate. Int J Biol Macromol.

[21] Eleftheriadis T, Pissas G, Liakopoulos V, Stefanidis I (2016). Cytochrome c as a potentially clinical useful marker of mitochondrial and cellular damage. Front Immunol.

[22] Dela Cruz CS, Kang MJ (2018). Mitochondrial dysfunction and damage associated molecular patterns (DAMPs) in chronic inflammatory diseases. Mitochondrion.

[23] Zhang M, Zheng J, Nussinov R, Ma B (2017). Release of cytochrome C from Bax pores at the mitochondrial membrane. Sci Rep.

[24] Campbell GR, To RK, Spector SA. TREM-1 Protects HIV-1-Infected Macrophages from Apoptosis through Maintenance of Mitochondrial Function. mBio. 2019;10(6):e02638–19. 10.1128/mBio.02638-19. 10.1128/mBio.02638-19 PMC6851287 31719184

[25] Wolter KG, Hsu YT, Smith CL, Nechushtan A, Xi XG, Youle RJ (1997). Movement of Bax from the cytosol to mitochondria during apoptosis. J Cell Biol.

[26] Kinnally KW, Antonsson B (2007). A tale of two mitochondrial channels, MAC and PTP, in apoptosis. Apoptosis.

[27] Fischer TW, Zmijewski MA, Wortsman J, Slominski A (2008). Melatonin maintains mitochondrial membrane potential and attenuates activation of initiator (casp-9) and effector caspases (casp-3/casp-7) and PARP in UVR-exposed HaCaT keratinocytes. J Pineal Res.

[28] Slominski A, Pisarchik A, Johansson O, Jing C, Semak I, Slugocki G (2003). Tryptophan hydroxylase expression in human skin cells. Biochim Biophys Acta.

[29] Tan DX, Manchester LC, Qin L, Reiter RJ. Melatonin: A Mitochondrial Targeting Molecule Involving Mitochondrial Protection and Dynamics. Int J Mol Sci. 2016;17(12):2124. 10.3390/ijms17122124. 10.3390/ijms17122124 PMC5187924 27999288

[30] Kim MK, Park EA, Kim HJ, Choi WY, Cho JH, Lee WS (2013). Does supplementation of in-vitro culture medium with melatonin improve IVF outcome in PCOS?. Reprod BioMed Online.

[31] Yu F, Zeng H, Lei M, Xiao DM, Li W, Yuan H (2016). Effects of SIRT1 gene knock-out via activation of SREBP2 protein-mediated PI3K/AKT signaling on osteoarthritis in mice. J Huazhong Univ Sci Technol Med Sci.

[32] Li XH, Chen C, Tu Y, Sun HT, Zhao ML, Cheng SX (2013). Sirt1 promotes axonogenesis by deacetylation of Akt and inactivation of GSK3. Mol Neurobiol.

[33] Gong S, Peng Y, Jiang P, Wang M, Fan M, Wang X (2014). A deafness-associated tRNAHis mutation alters the mitochondrial function, ROS production and membrane potential. Nucleic Acids Res.

[34] Liu CW, Yang F, Cheng SZ, Liu Y, Wan LH, Cong HL (2017). Rosuvastatin postconditioning protects isolated hearts against ischemia-reperfusion injury: the role of radical oxygen species, PI3K-Akt-GSK-3β pathway, and mitochondrial permeability transition pore. Cardiovasc Ther.

[35] Rohlenova K, Neuzil J, Rohlena J (2016). The role of Her2 and other oncogenes of the PI3K/AKT pathway in mitochondria. Biol Chem.

[36] Simonyan L, Renault TT, Novais MJ, Sousa MJ, Côrte-Real M, Camougrand N (2016). Regulation of Bax/mitochondria interaction by AKT. FEBS Lett.

[37] Escobar-Morreale HF, San Millán JL (2007). Abdominal adiposity and the polycystic ovary syndrome. Trends Endocrinol Metab.

[38] Spritzer PM, Lecke SB, Satler F, Morsch DM (2015). Adipose tissue dysfunction, adipokines, and low-grade chronic inflammation in polycystic ovary syndrome. Reproduction.

