# Supplementary material for: Melatonin enhances SIRT1 to ameliorate mitochondrial membrane damage by activating PDK1/Akt in granulosa cells of PCOS
Source: J Ovarian Res. 2021 Nov 11;14:152. doi: 10.1186/s13048-021-00912-y (PMC8582167; doi:10.1186/s13048-021-00912-y)

**Supplementary Figure 1. Raw data of western blotting**

1. **p-Akt of human samples in Fig.1**


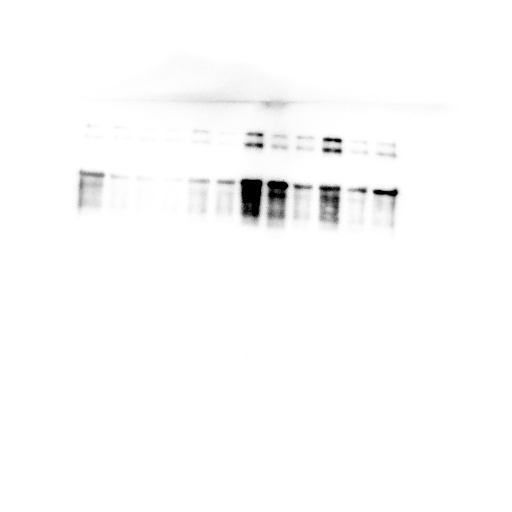


1. **Akt of human samples in Fig.1**


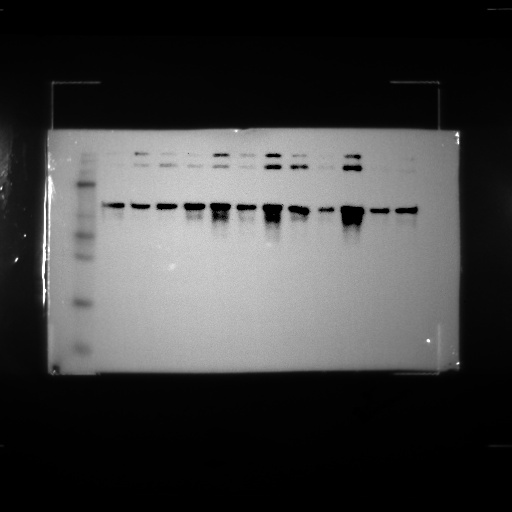


1. **Beta-actin of human samples in Fig.1**


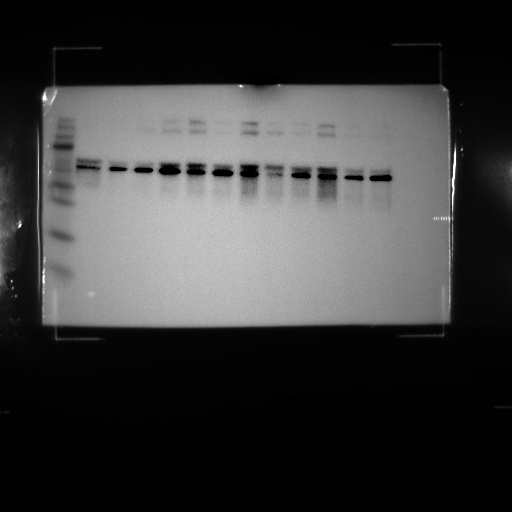


1. **Cytochrome C of mouse samples in Fig.2**


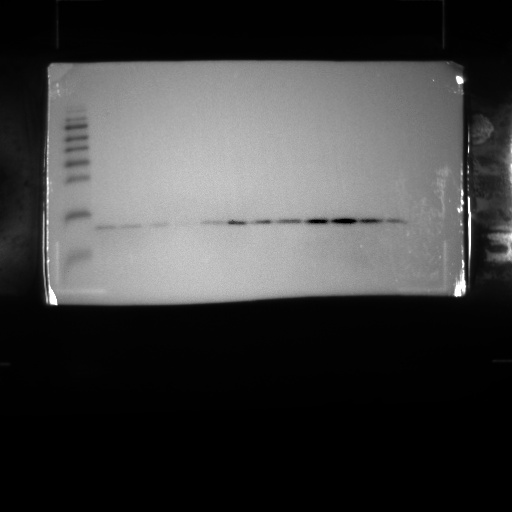


1. **Beta-actin of mouse samples in Fig.2**

**
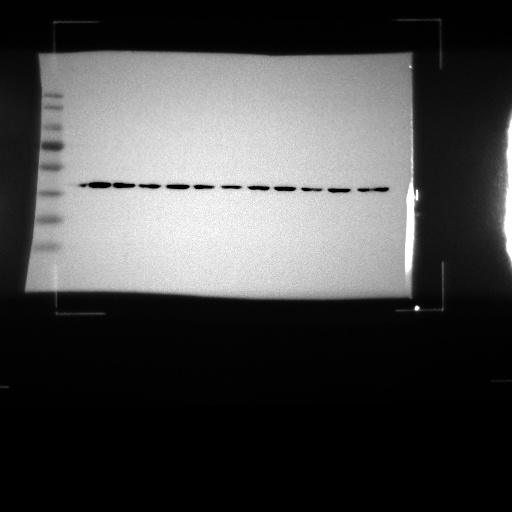
**

1. **BCL2 of mouse samples in Fig.2**

**
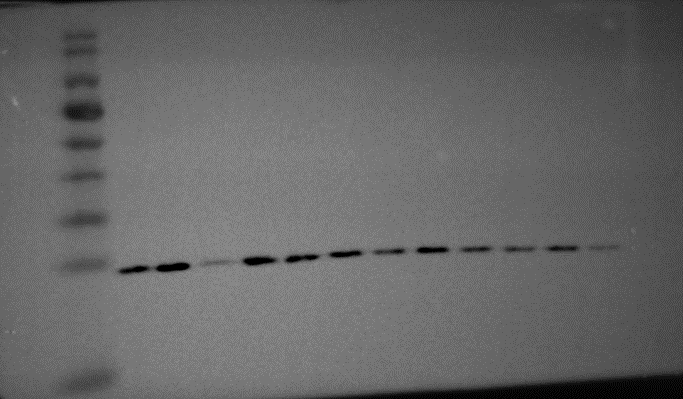
**

1. **BAX of mouse samples in Fig.2**


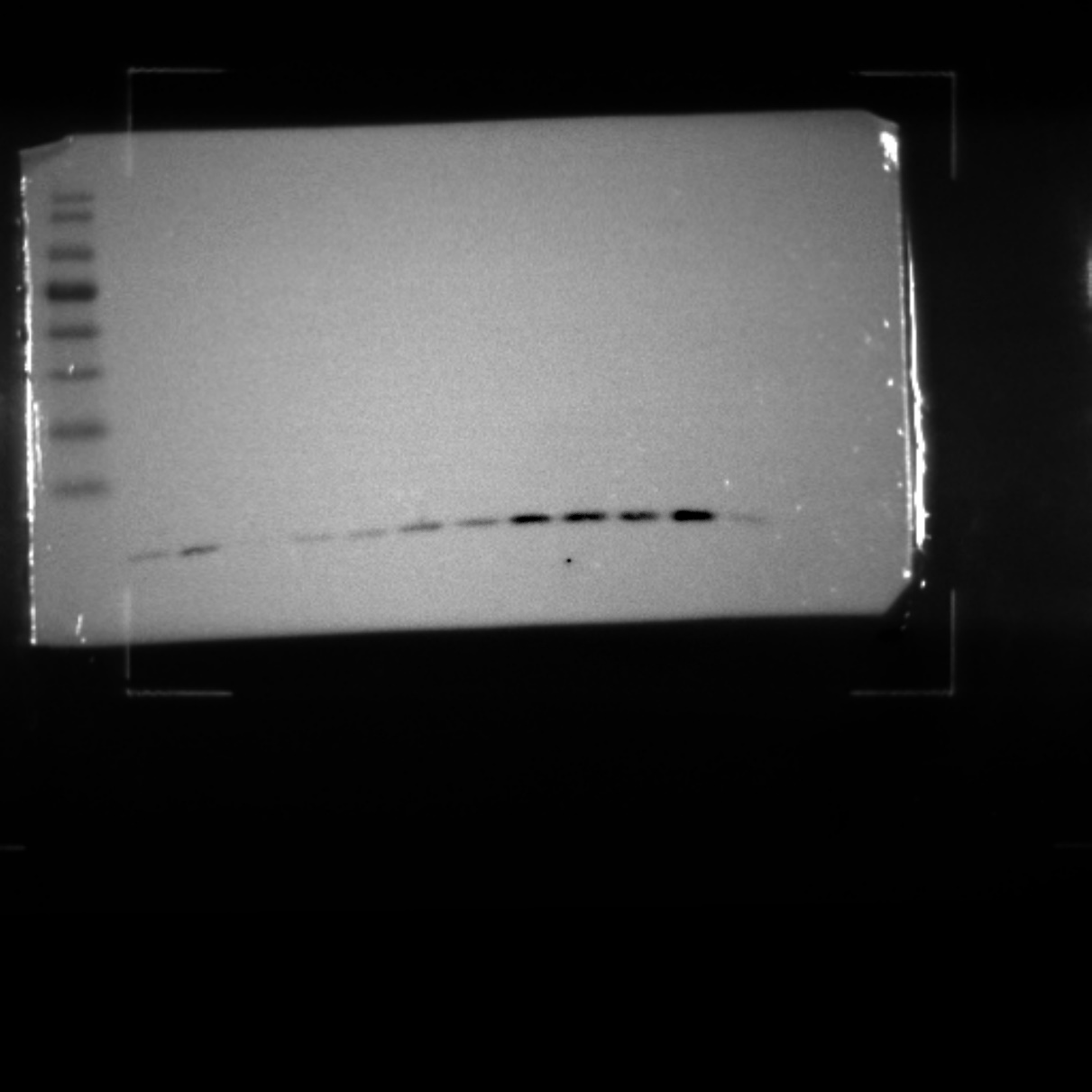


1. **VDAC1 of mouse samples in Fig.2**


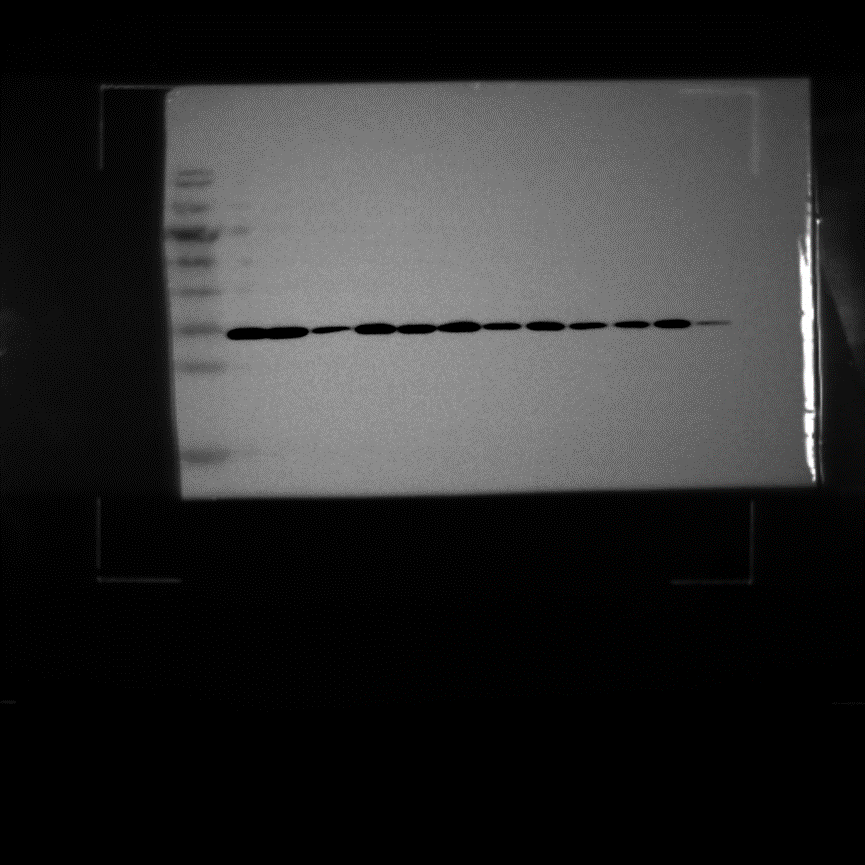


1. **p-AKT of KGN cell samples in Fig.3**


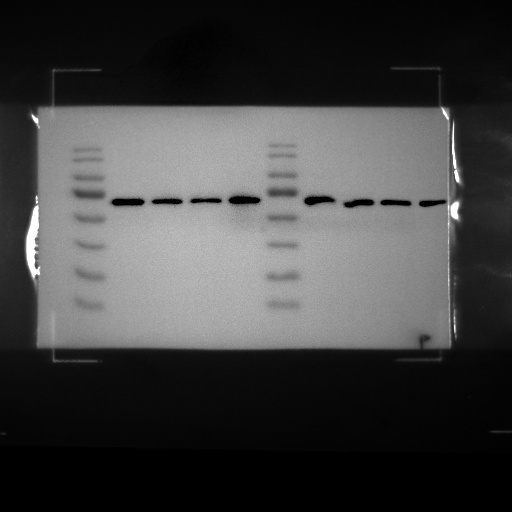


1. **AKT of KGN cell samples in Fig.3**

**
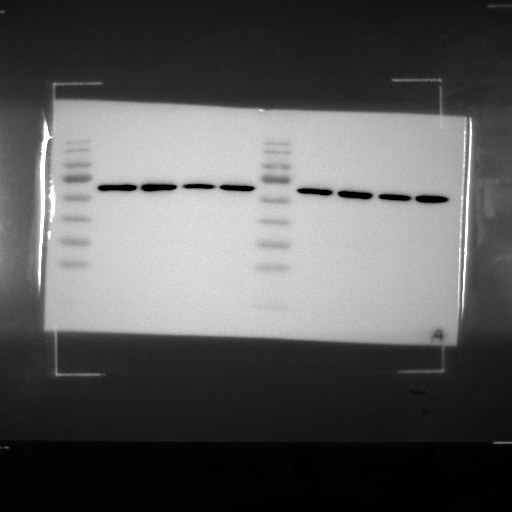
**

1. **Beta-actin of KGN cell samples in Fig.3**


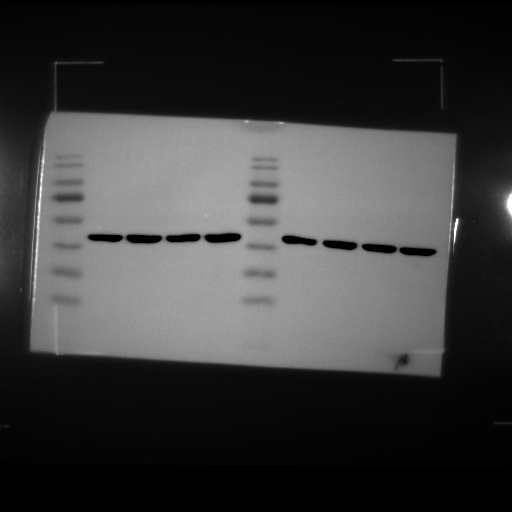


**Supplementary Figure 2.**

1. **Cycle stages of all PCOS mice and control mice**


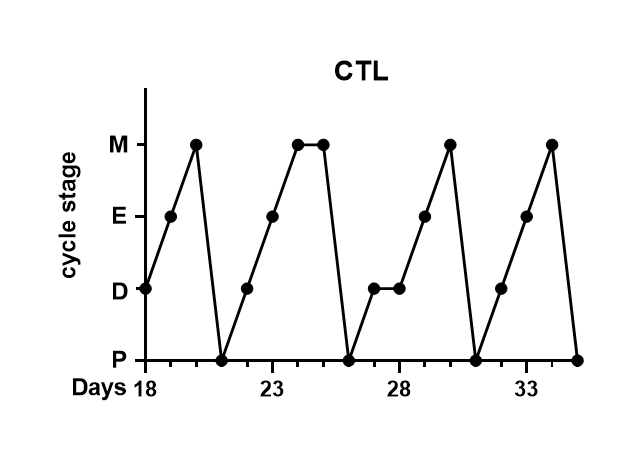

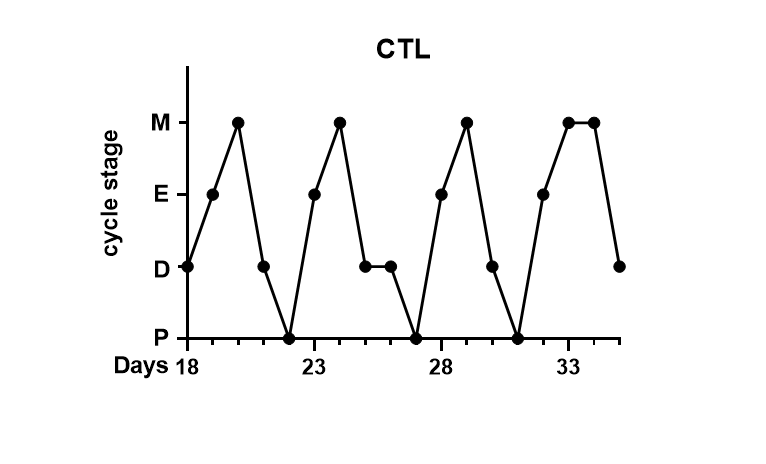


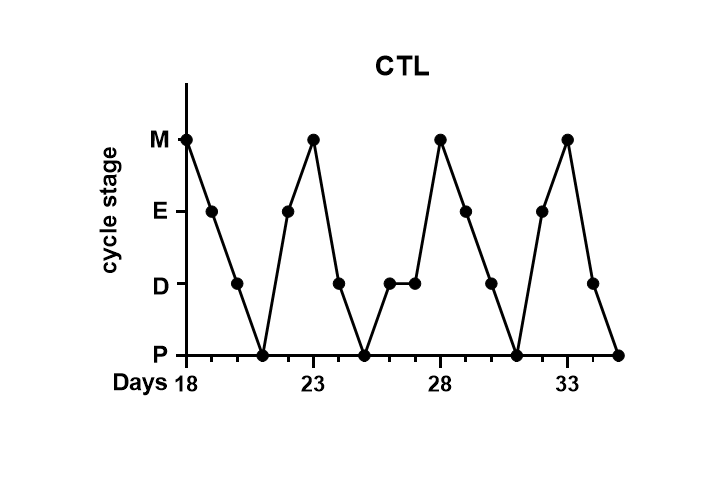

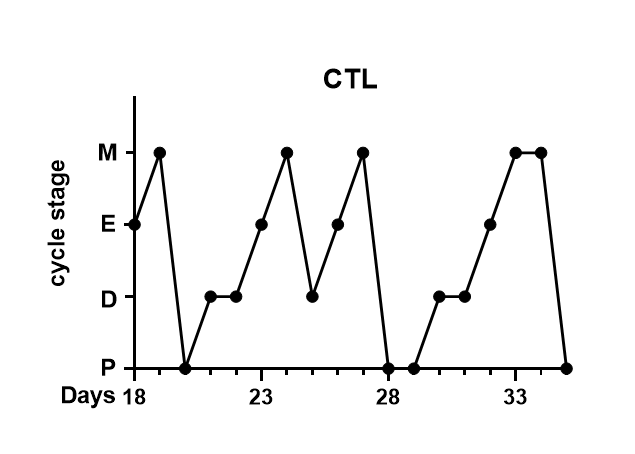


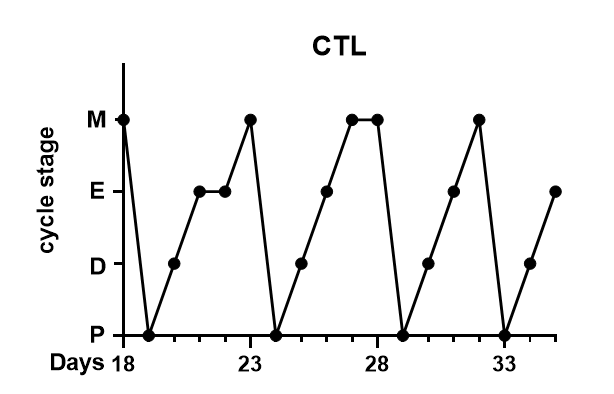

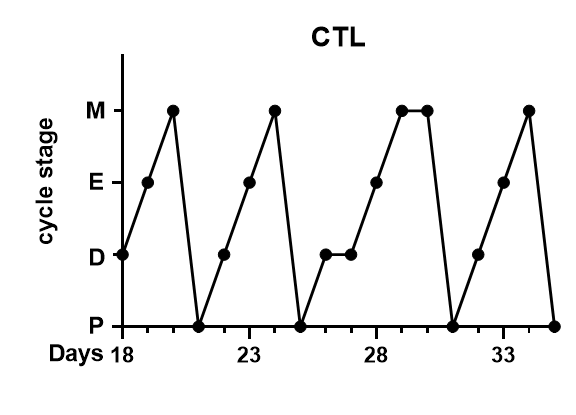


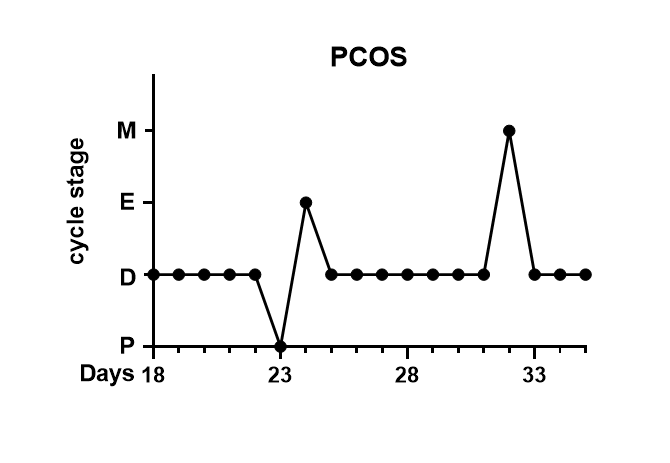

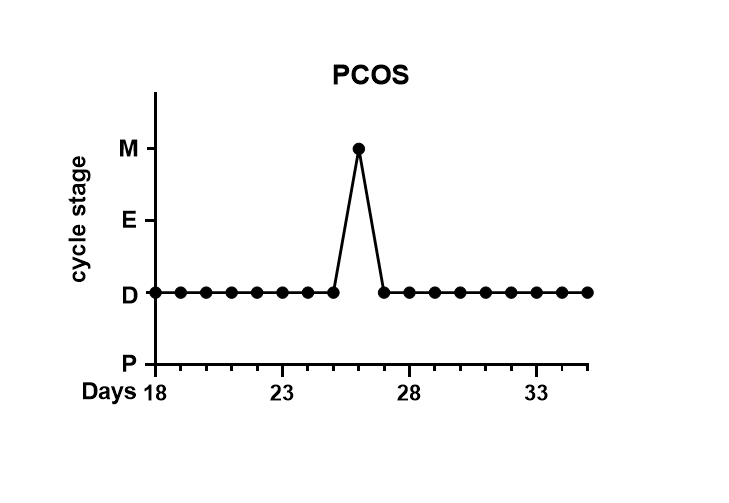


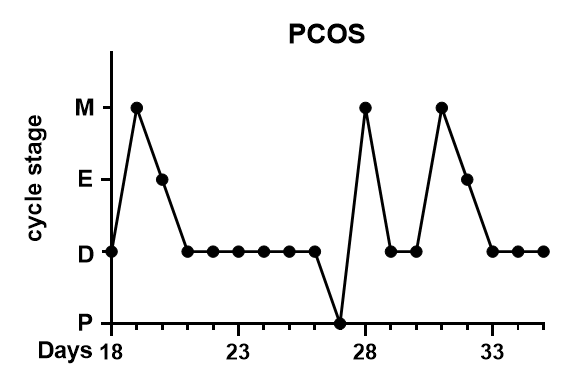

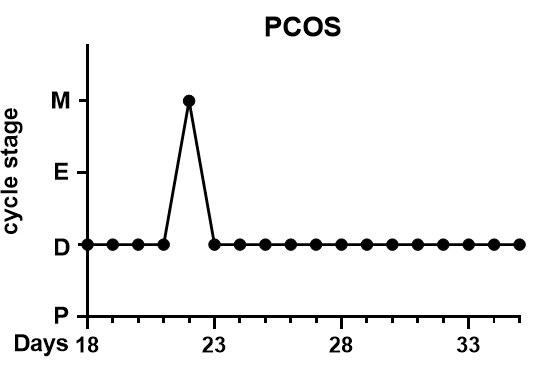


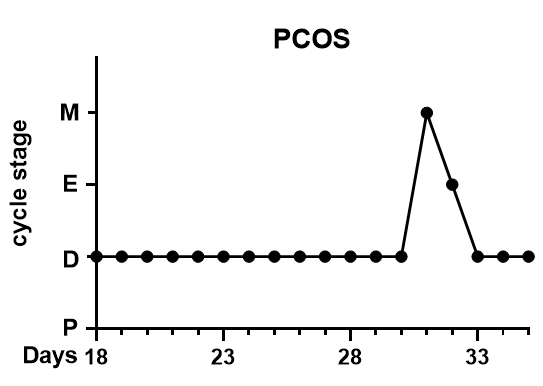

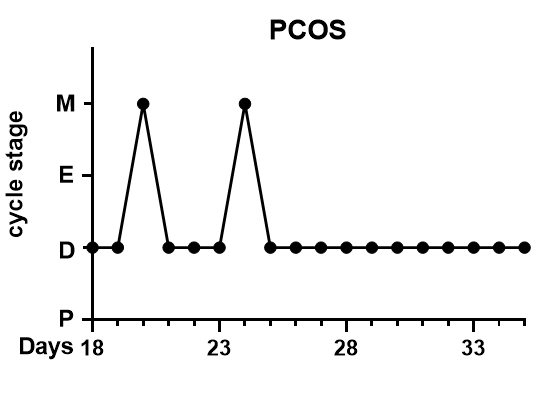


**Supplementary Figure 3.**

1. **Expression of Beta-actin between PCOS and Control in Fig 1**


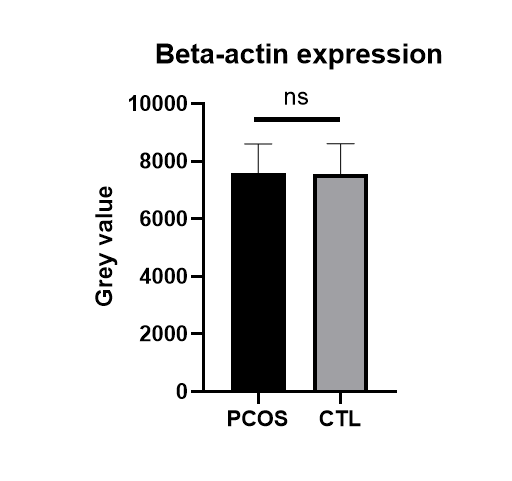

Supplement: Supplementary file 1 — Additional file 1: Supplementary Figure 1. Raw data of western blotting. Supplementary Figure 2. Cycle stages of all PCOS mice and control mice. Supplementary Figure 3. Expression of Beta-actin between PCOS and Control in Fig. 1 [file 13048_2021_912_MOESM1_ESM.docx]
